# KSHV co-infection down-regulates HPV16 E6 and E7 from cervical cancer cells

**DOI:** 10.18632/oncotarget.16207

**Published:** 2017-03-15

**Authors:** Lu Dai, Yueyu Cao, Wei Jiang, Jovanny Zabaleta, Zhongmin Liu, Jing Qiao, Zhiqiang Qin

**Affiliations:** ^1^ Department of Pediatrics, East Hospital, Tongji University School of Medicine, Shanghai 200120, China; ^2^ Research Center for Translational Medicine and Key Laboratory of Arrhythmias, East Hospital, Tongji University School of Medicine, Shanghai 200120, China; ^3^ Department of Microbiology and Immunology, Division of Infectious Diseases, Department of Medicine, Medical University of South Carolina, Charleston, SC 29425, USA; ^4^ Departments of Genetics Louisiana State University Health Sciences Center, Louisiana Cancer Research Center, New Orleans, LA 70112, USA; ^5^ Pediatrics, Louisiana State University Health Sciences Center, Louisiana Cancer Research Center, New Orleans, LA 70112, USA

**Keywords:** HPV, KSHV, E6, E7, microRNA

## Abstract

High-risk human papillomavirus (HPV) infection is the etiological agent of some malignancies such as cervical, oral and oropharyngeal cancers. Kaposi sarcoma-associated herpesvirus (KSHV) represents a principal causative agent of several human cancers arising in those immunocompromised patients. Interestingly, KSHV DNA has been detected in the oral cavity and the female genital tract, although its detection rate in cervical samples is very low and few reports are about KSHV/HPV co-infection. Therefore, it remains unclear about the role of KSHV co-infection in the development of HPV-related neoplasias. In the current study, we report that HPV16-integrated cervical cancer cell-line SiHa is susceptible to KSHV latent infection and replication. We also have found that KSHV infection or viral latent proteins are capable of reducing HPV16 E6/E7 expression through the manipulation of cellular microRNA function. Array analysis indicates that KSHV infection induces some inflammatory cytokines/chemokines production as well as up-regulates a series of interferon-induced genes expression, which may facilitate host immune defense system attacking these co-infected cells and clearance of viruses. Together, our data have provided possible explanations for very low detection rate of KSHV shedding as well as of KSHV/HPV co-infection in cervical samples and/or cervical cancer cells.

## INTRODUCTION

Cervical cancer represents one of the most common malignancies in females worldwide. The pathogenesis of cervical cancer occurs following persistent infection with high-risk human papillomavirus (HPV) such as subtype 16 and 18 [1]. High-risk HPV-encoded E6 and E7 proteins are major viral oncoproteins which are closely associated with human cervical carcinogenesis [2]. E6 and E7 can bind to the p53 and retinoblastoma (Rb) family proteins, respectively, resulting in the regulation of cell cycle and final transformation [3]. In addition, high-risk HPV infection is also prevalent in oral cavity and related to oral and oropharyngeal cancer development [4–6].

Another oncogenic virus, Kaposi sarcoma-associated herpesvirus (KSHV) represents a principal causative agent of several human cancers arising in those immunocompromised patients, including Kaposi's Sarcoma (KS), Primary effusion lymphoma (PEL) and Multicentric Castleman's disease (MCD) [7–9]. Published literatures have reported that KSHV DNA sequences can be detected in the prostate [10], semen [11], oral cavity [12] and the female genital tract [13, 14]. Moreover, person-to-person transmission of KSHV is thought to occur primarily through exchange of oropharyngeal secretions [15]. In contrast to the high prevalence of KSHV shedding in oral cavity, the detection rate of KSHV DNA or virus infection in cervical samples are relatively low (< 2%), even in those high-risk population such as sex workers and HIV+ persons [14, 16]. Furthermore, currently there are few studies reporting the co-infection of KSHV and HPV in cervical samples and/or cervical cancer cells. Therefore, it remains unclear about the role of KSHV co-infection in the development of HPV-related neoplasias. In the current study, we tested the susceptibility of HPV16-integrated cervical cancer cell-line SiHa to KSHV infection, replication and their impacts on HPV-encoded oncoproteins expression. Interestingly, we found that KSHV *de novo* infection or viral latent proteins significantly reduced HPV16 E6/E7 expression through the manipulation of cellular microRNA function. We also found that KSHV infection induced some inflammatory cytokines/chemokines production as well as up-regulated a series of interferon-induced genes expression, which may facilitate host immune defense system attacking these co-infected cells and clearance of viruses.

## RESULTS

### KSHV can establish latent infection within SiHa cells and possess replicative potential

**Figure 1:**
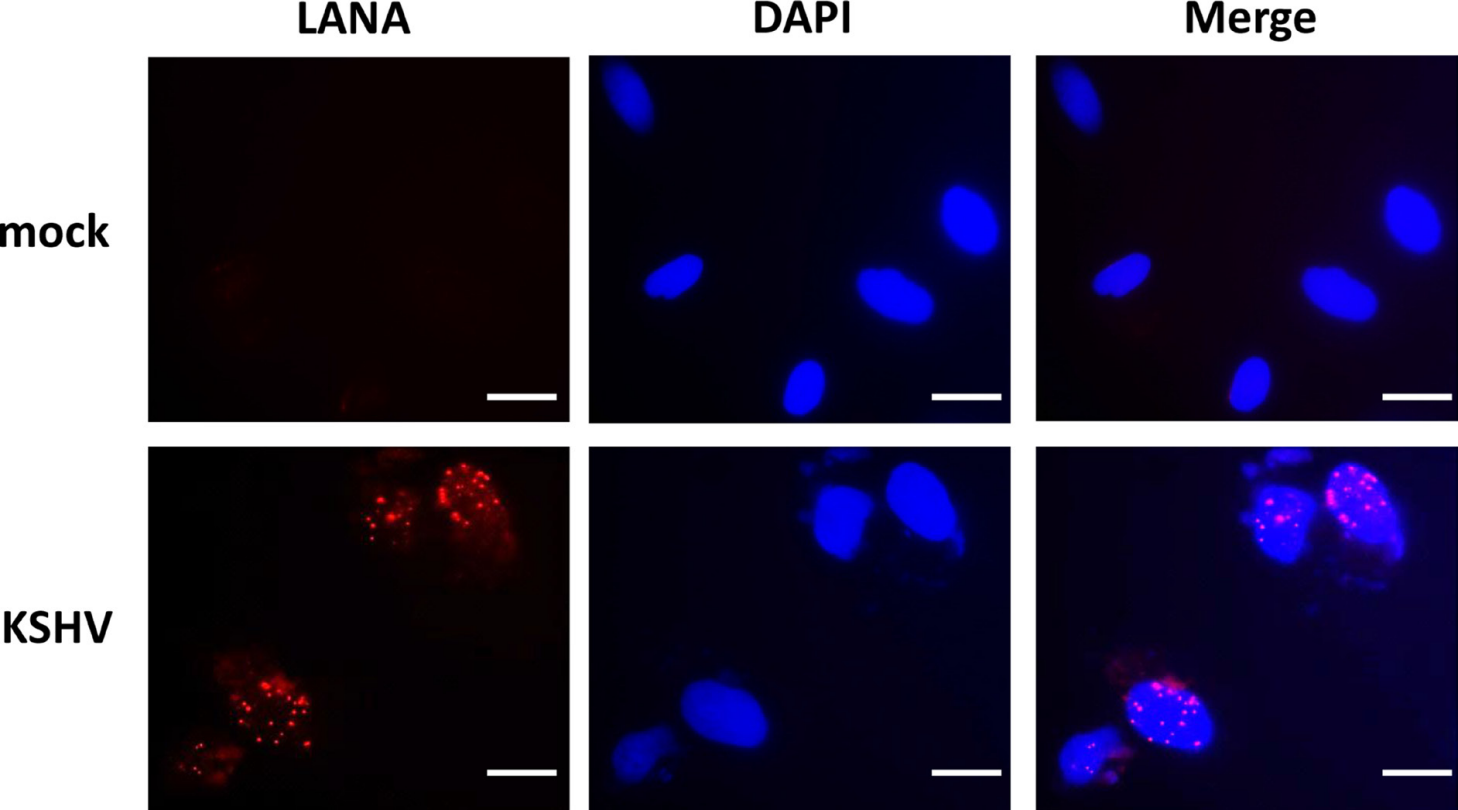
Establishment of latent KSHV infection within SiHa cells SiHa were incubated with purified KSHV (MOI~10), or medium control (mock) for 2 h. After cells were incubated for an additional 72 h in fresh media, immunofluorescence was performed to quantify expression of KSHV-encoded LANA as indicated by the typical intranuclear, punctate staining pattern (red dots). Nuclei were identified using DAPI (blue). Bars, 20 μm.

**Figure 2:**
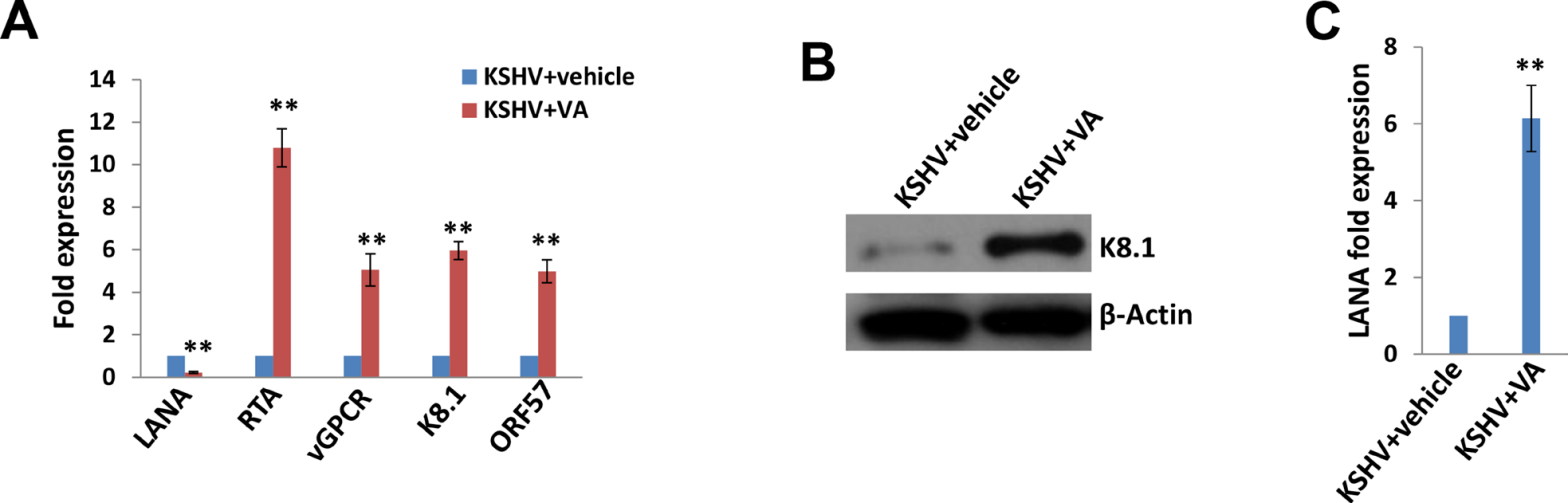
Induction of lytic reactivation and virus production from KSHV latently infected SiHa cells (**A**–**B**) KSHV latently infected SiHa cells were incubated with 0.6 mM valproic acid (VA) or vehicle for 5 days, then qRT-PCR and immunoblots were performed as described in the Methods. (**C**) The virion production was collected as described in the Methods, followed by infection of fresh SiHa cells. Lana transcripts were quantified by using qRT-PCR. Error bars represent the S.D. for 3 independent experiments, **= *p* < 0.01.

Latent KSHV infection is dependent upon intranuclear expression of KSHV-encoded latency associated nuclear antigen (LANA), which tethers viral episome to host cell chromatin [17]. To first determine whether SiHa cells are susceptible to KSHV infection, we incubated them with purified KSHV virions and used immunofluorescence (IFA) to quantify LANA expression within individual cells. The confocal microscopy images revealed LANA expression within > 95% of SiHa cell nuclear following 72 h post infection (p.i.) with an MOI ~10, while no LANA dots observed in the control mock cells (Figure 1). To further validate replicative potential of these viruses in latently infected SiHa cells, we treated infected cells with valproic acid (VA) for 5 days, a common chemical inducing viral lytic reactivation [18]. Then we used qRT-PCR to quantify the transcripts of representative viral latent and lytic genes. Our results indicated VA treatment greatly induced different viral lytic genes expression (RTA, vGPCR, K8.1 and ORF57), while dramatically reducing latent gene LANA expression from infected SiHa cells (Figure 2A). Immunoblots analysis confirmed the elevated expression of K8.1, one of lytic proteins by VA (Figure 2B). Furthermore, we found that VA induced infected SiHa cells release of infectious KSHV particles in culture supernatants, as demonstrated by increased LANA expression within fresh KSHV-naïve cells following their exposure to VA-treated SiHa supernatants (Figure 2C). Together, our data demonstrate that SiHa are susceptible to KSHV latent infection and these viruses are capable of self-replication once stimulated to lytic reactivation.

### Global signature of cellular cytokine/chemokine altered within KSHV-infected SiHa cells

**Figure 3:**
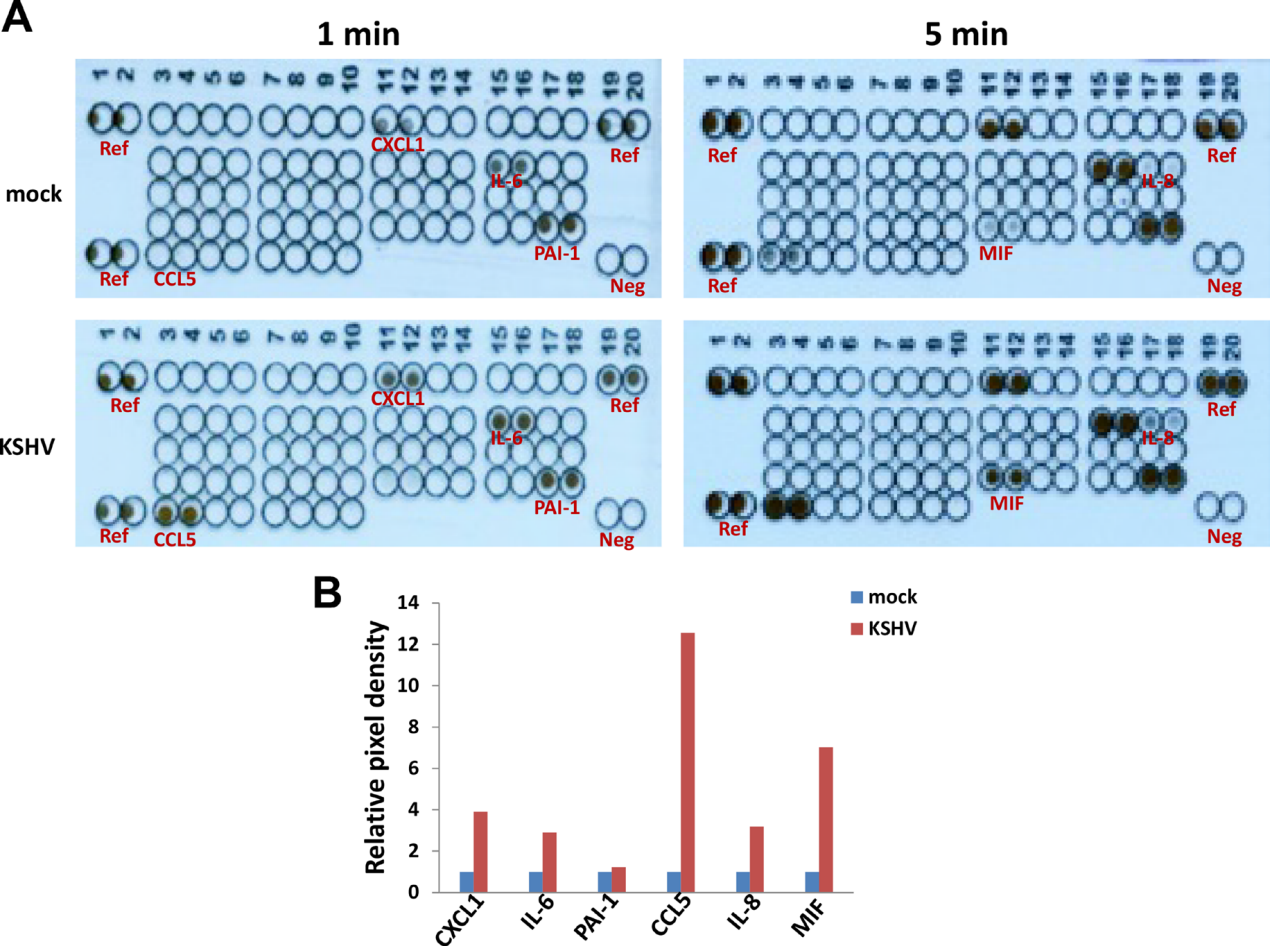
Cytokine/chemokine profile altered within KSHV latently infected SiHa cells (**A**) SiHa were incubated with purified KSHV (MOI ~ 10), or medium control (mock) as describe above, then the supernatants were collected and the concentrations of different cytokines/chemokines were measured as described in the Methods. (**B**) The density of dot-blot was scanned and quantified by using the ImageJ software. Ref: reference positive control wells; Neg: negative control wells.

By using a cytokine/chemokine array, we identified a global signature altered within KSHV-infected SiHa when compared to the control mock cells. We found that KSHV infection increased several inflammatory factors production from SiHa cells, including Chemokine (C-X-C motif) ligand 1 (CXCL1), Interleukin 6 (IL-6), Plasminogen activator inhibitor-1 (PAI-1), Chemokine (C-C motif) ligand 5 (CCL5), Interleukin 8 (IL-8) and Macrophage migration inhibitory factor (MIF) (Figure 3). Our additional data have demonstrated KSHV infection does not affect SiHa cell growth and viability (Supplementary Figure 1), therefore which is not responsible for the increased cytokines/chemokines production we have observed.

### The down-regulation of HPV16 E6 and E7 by KSHV and/or viral latent proteins

**Figure 4:**
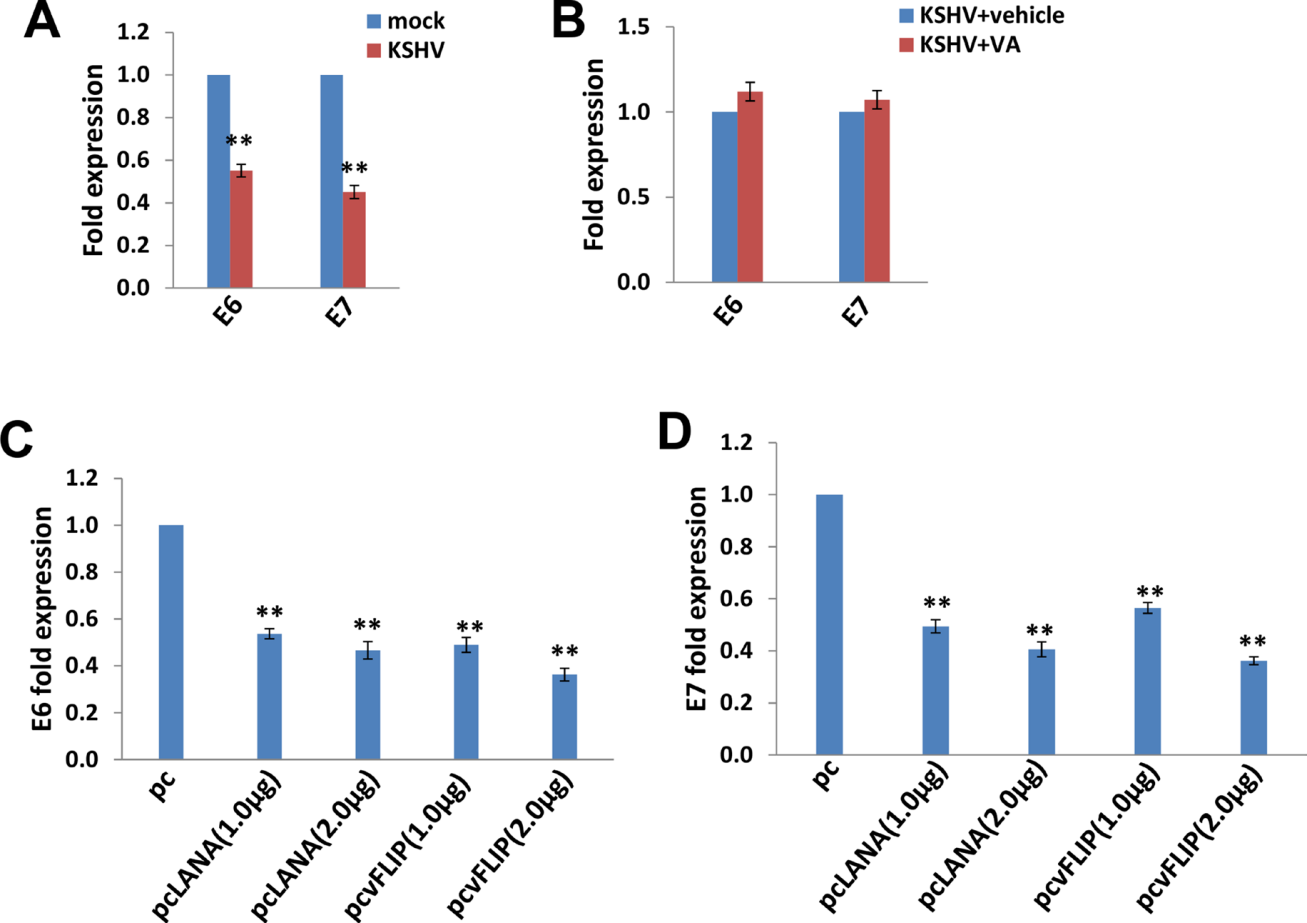
The down-regulation of HPV16 E6 and E7 by KSHV and/or viral latent proteins (**A**–**B**) SiHa cells were infected by KSHV or induced lytic reactivation as described above, then gene transcripts were quantified by using qRT-PCR. (**C**–**D**) SiHa were transfected with control vector pcDNA3.1 (pc), pcDNA3.1-LANA (pcLANA) or pcDNA3.1-vFLIP (pcvFLIP) as described in the Methods, then gene transcripts were quantified by using qRT-PCR. The Error bars represent the S.D. for 3 independent experiments, **= *p* < 0.01.

SiHa cells contain an integrated HPV16 genome, and E6/E7 represent major HPV-encoded oncogenic proteins [1, 2]. Interestingly, our data indicated that KSHV infection significantly reduced both E6 and E7 expression from SiHa cells (Figure 4A). In contrast to this, VA treatment abolished this reduction, implying the important role of KSHV-encoded latent proteins in this regulation (Figure 4B). To prove that, we ectopically expressed LANA and vFLIP (viral FLICE inhibitory protein), two major KSHV-encoded latent proteins in SiHa cells using the recombinant constructs, respectively. We found that ectopic expression of either LANA or vFLIP effectively down-regulated E6 and E7 expression in a dose-dependent manner (Figure 4C–4D).

### The up-regulation of miR129-5p is required for KSHV/viral proteins reducing E6 and E7 expression

**Figure 5:**
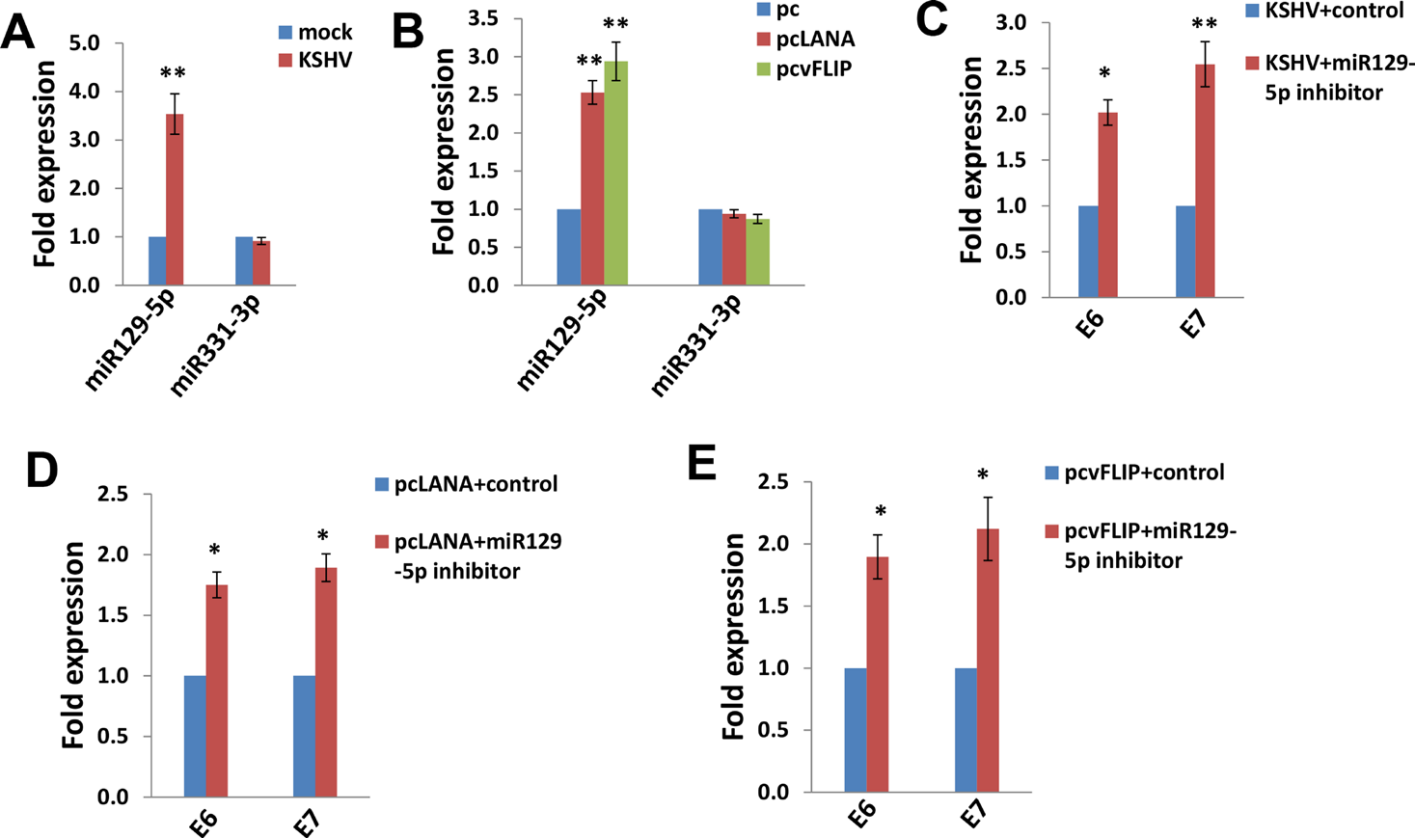
The up-regulation of miR129-5p is required for KSHV/viral latent proteins reducing HPV16 E6 and E7 expression (**A**–**B**) SiHa cells were infected by KSHV or transfected using LANA or vFLIP recombinant constructs as described above, then miRNAs transcripts were quantified by using qRT-PCR. (**C**–**E**) SiHa cells were transfected with control or miR129-5p inhibitor prior to KSHV infection or ectopic expression of LANA or vFLIP, then gene transcripts were quantified by using qRT-PCR. The Error bars represent the S.D. for 3 independent experiments, *= *p* < 0.05, **= *p* < 0.01.

We subsequently sought to determine how KSHV infection and/or viral latent proteins reducing E6 and E7 expression. One of potential mechanisms is through cellular microRNAs such as miRNA129-5p and miRNA331-3p [19, 20]. Zhang *et al*. have reported that interferon-β treatment can induce miRNA129-5p, while its levels gradually decrease with the development of cervical intraepithelial lesions and correlate with E6 and E7 expression [19]. Another group has found that miRNA331-3p can suppress cervical cancer cell proliferation and E6/E7 expression by targeting Neuropilin 2 (NRP2) [20]. So we tested these 2 cellular microRNAs expression in SiHa cells. We found that either KSHV infection or ectopic expression of LANA or vFLIP significantly increased miRNA129-5p but not miRNA331-3p from SiHa cells (Figure 5A–5B). We next used specific miRNA129-5p inhibitor to block its activities (Supplementary Figure 2), which effectively restored E6/E7 expression from KSHV-infected or LANA-/vFLIP-transfected SiHa cells (Figure 5C–5E). These data demonstrate that miRNA129-5p but not miRNA331-3p is required for KSHV and/or viral latent proteins reducing E6/E7 expression from SiHa cells.

### Transcriptomic analysis of the gene profile altered in KSHV-infected SiHa cells

**Figure 6:**
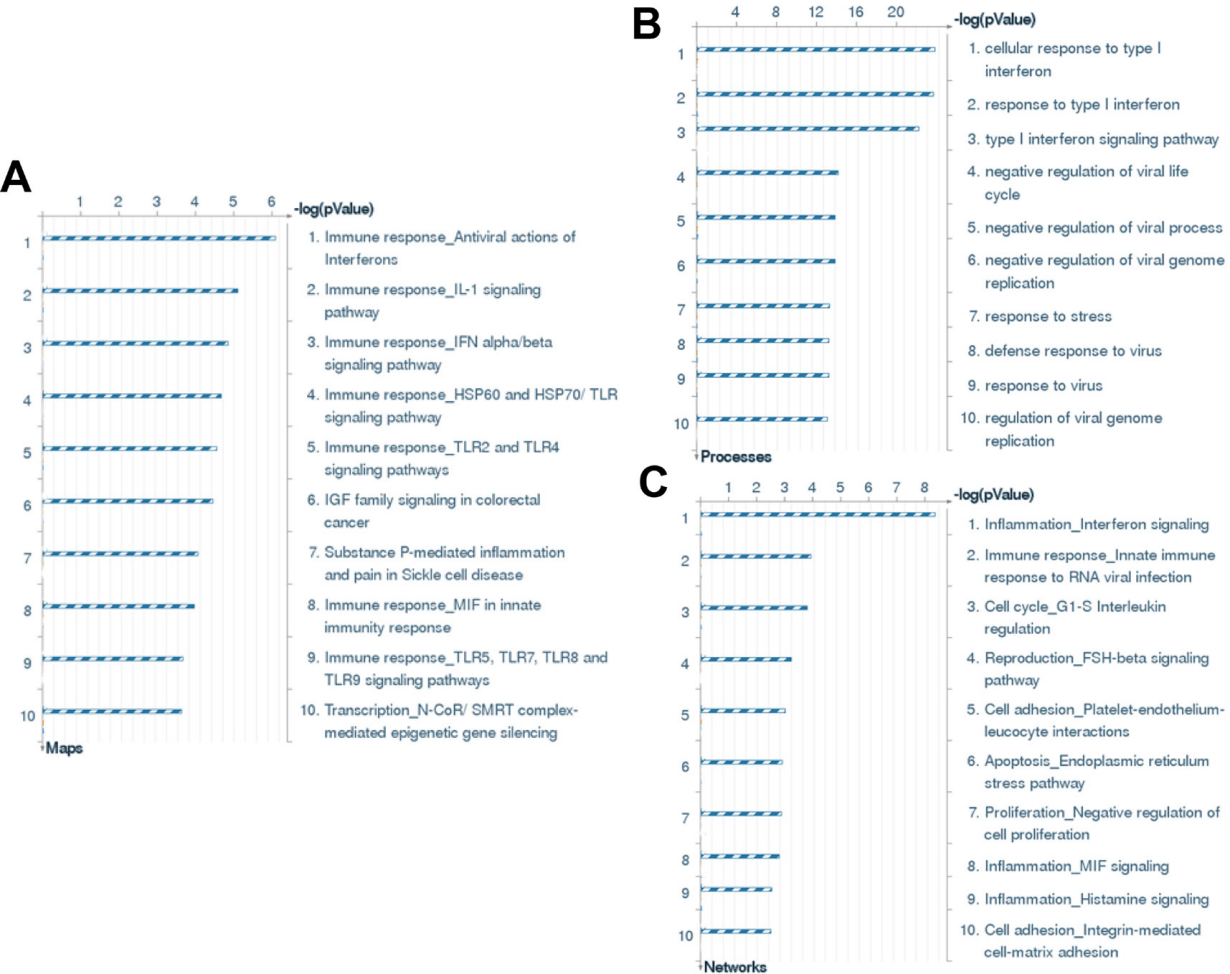
The enrichment analysis of gene profile alterations in KSHV-infected SiHa cells (**A**–**C**) The enrichment analysis of gene profile significantly altered (up/down ≥ 2 fold and *p* < 0.05) in KSHV-infected SiHa cells was performed using the Metacore Software (Thompson Reuters) Modules: Pathway Maps (A), Gene Ontology Processes (B), and Process Networks (C).

**Figure 7:**
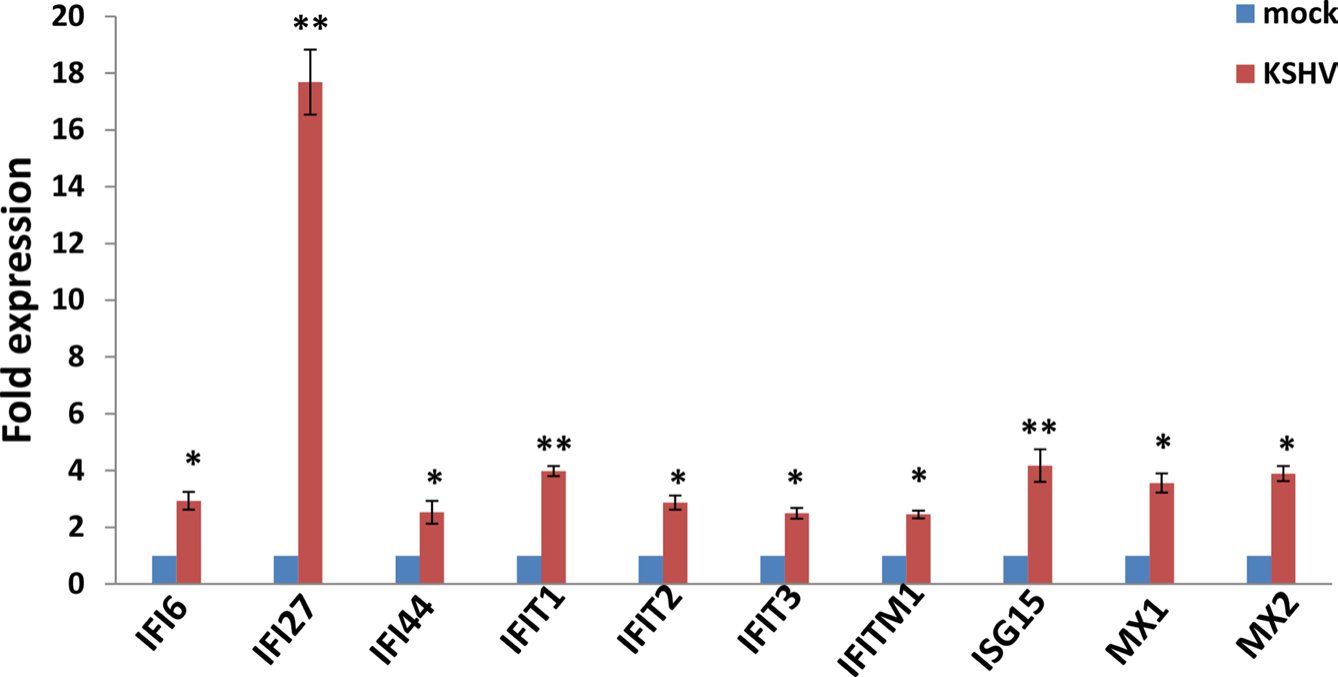
The up-regulation of interferon-induced genes from KSHV-infected SiHa cells SiHa cells were infected by KSHV as described above, then gene transcripts were quantified by using qRT-PCR. The Error bars represent the S.D. for 3 independent experiments, *= *p* < 0.05, **= *p* < 0.01.

We used the HumanHT-12 v4 Expression BeadChip (Illumina), which contains more than 47,000 probes derived from the NCBI RefSeq Release 38 and other sources, to study the gene profile altered within KSHV-infected SiHa cells. We found that 71 genes were significantly up-regulated and 27 were down-regulated (≥ 2 fold and *p* < 0.05) within KSHV-infected SiHa cells when compared to the control mock cells (Tables 1 and 2). For validation of microarray analysis, we next selected 5 candidate genes from Tables 1 and 2, respectively, to perform qRT-PCR analysis. Our results indicated that all of the 10 selected genes were significantly altered in a manner comparable to those found in the microarray data, demonstrating the credibility of our results. Specifically, *IFI27*, *KRT34*, *IL6*, *CEMIP* and *MX1* were significantly up-regulated, while *AKR1C3*, *SEMA3C*, *ATOH8*, *ALDH3A1* and *CRIP2* were significantly down-regulated within KSHV-infected SiHa cells (Supplementary Figure 3). We also performed enrichment analysis of these significantly altered candidates by using the Pathway Maps, Gene Ontology (GO) Processes and Process Networks modules from Metacore Software (Thompson Reuters). Our analysis showed that these significantly altered candidates belong to several major functional categories, including cellular response to type I interferon (IFN), defense response to virus infection/replication, cellular response to stress and inflammation (Figure 6). In addition, the top 2 scored pathway maps and protein networks were also listed in Supplementary Figures 4 and 5, respectively. Notably, we found that KSHV infection induced strong cellular response to type I IFN, especially those IFN-induced genes expression. Our qRT-PCR data confirmed that a series of 10 IFN-induced genes were significantly up-regulated from KSHV-infected SiHa cells when compared to the control mock cells (Figure 7).

**Table 1:** The genes up-regulated within KSHV-infected SiHa cells

Gene Symbol	Description	Ratio
IFI27	Interferon alpha-inducible protein 27, mitochondrial	13.24683
KRT34	Keratin, type I cuticular Ha4	10.96868
IL6	Interleukin-6	6.189084
CEMIP	Cell migration-inducing and hyaluronan-binding protein	4.637684
MX2	Interferon-induced GTP-binding protein Mx2	4.297035
EPSTI1	Epithelial-stromal interaction protein 1	3.975506
RSAD2	Radical S-adenosyl methionine domain-containing protein 2	3.695813
MX1	Interferon-induced GTP-binding protein Mx1	3.616665
DHRS2	Dehydrogenase/reductase SDR family member 2, mitochondrial	3.584949
CXCL8	Interleukin-8	3.533108
HIST1H4H	Histone H4	3.443781
NT5E	5′-nucleotidase	3.408309
ISG15	Ubiquitin-like protein ISG15	3.371505
IGF2BP2	Insulin-like growth factor 2 mRNA-binding protein 2	3.326956
NKD2	Protein naked cuticle homolog 2	3.230526
GADD45A	Growth arrest and DNA damage-inducible protein GADD45 alpha	3.21191
OASL	2′-5′-oligoadenylate synthase-like protein	3.158974
USP18	Ubl carboxyl-terminal hydrolase 18	3.0996
STC2	Stanniocalcin-2	2.99668
RASD1	Dexamethasone-induced Ras-related protein 1	2.964571
LINC00161	long intergenic non-protein coding RNA 161 transcript	2.940959
IFIT1	Interferon-induced protein with tetratricopeptide repeats 1	2.915374
PPP1R15A	Protein phosphatase 1 regulatory subunit 15A	2.883852
IFIT2	Interferon-induced protein with tetratricopeptide repeats 2	2.849808
IFI44	Interferon-induced protein 44	2.807813
RELB	Transcription factor RelB	2.803865
AP-1	Transcription factor AP-1	2.746863
PLD6	Mitochondrial cardiolipin hydrolase	2.714222
SNHG15	small nucleolar RNA host gene 15 transcript	2.642434
PHLDA1	Pleckstrin homology-like domain family A member 1	2.604317
IFIT3	Interferon-induced protein with tetratricopeptide repeats 3	2.598199
RIMKLB	Beta-citrylglutamate synthase B	2.581286
OAS1	2′-5′-oligoadenylate synthetase 1	2.571998
LRP5L	Low-density lipoprotein receptor-related protein 5-like protein	2.488861
CCND1	G1/S-specific cyclin-D1	2.451345
DUSP5	Dual specificity protein phosphatase 5	2.441991
OLR1	Oxidized low-density lipoprotein receptor 1	2.431704
LOC285074	Anaphase promoting complex subunit 1 pseudogene	2.369141
CCL5	C-C motif chemokine 5	2.359643
IFI6	Interferon alpha-inducible protein 6	2.327306
CTGF	Connective tissue growth factor	2.324625
SLCO2A1	Solute carrier organic anion transporter family member 2A1	2.321616
DDIT3	DNA damage-inducible transcript 3 protein	2.312649
MAFF	Transcription factor MafF	2.29661
OTUD1	OTU domain-containing protein 1	2.268293
SNHG12	Putative uncharacterized protein SNHG12	2.267007
TUBB2B	Tubulin beta-2B chain	2.262177
ETV5	ETS translocation variant 5	2.257368
PMAIP1	Phorbol-12-myristate-13-acetate-induced protein 1	2.244876
HIST1H4D	Histone H4	2.243342
WARS	Tryptophan--tRNA ligase, cytoplasmic	2.23195
PDP1	[Pyruvate dehydrogenase [acetyl-transferring]]-phosphatase 1, mitochondrial	2.217114
OAS2	2′-5′-oligoadenylate synthetase 2	2.199281
ASNS	Asparagine synthetase [glutamine-hydrolyzing]	2.19681
LINC01554	Putative uncharacterized protein encoded by LINC01554	2.158002
NR2C1	Nuclear receptor subfamily 2 group C member 1	2.154326
KRT16	Keratin, type I cytoskeletal 16	2.144548
EPB41L4A-AS1	EPB41L4A antisense RNA 1 transcript	2.143205
GADD45B	Growth arrest and DNA damage-inducible protein GADD45 beta	2.142809
HIST1H2BC	Histone H2B type 1-C/E/F/G/I	2.140187
SLC15A3	Solute carrier family 15 member 3	2.117546
ERRFI1	ERBB receptor feedback inhibitor 1	2.112755
HIST1H4E	Histone H4	2.108247
TAF1D	TATA box-binding protein-associated factor RNA polymerase I subunit D	2.108209
IFITM1	Interferon-induced transmembrane protein 1	2.096043
SNAPC4	snRNA-activating protein complex subunit 4	2.059977
ARNT2	Aryl hydrocarbon receptor nuclear translocator 2	2.028269
SNHG1	small nucleolar RNA host gene 1 transcript	2.01646
RHOB	Rho-related GTP-binding protein RhoB	2.015713
HELZ2	Helicase with zinc finger domain 2	2.015691
NFIL3	Nuclear factor interleukin-3-regulated protein	2.014101

**Table 2:** The genes down-regulated within KSHV-infected SiHa cells

Gene Symbol	Description	Ratio
AKR1C3	Aldo-keto reductase family 1 member C3	0.273224
KRT19	Keratin, type I cytoskeletal 19	0.297783
SBSPON	Somatomedin-B and thrombospondin type-1 domain-containing protein	0.339295
SEMA3C	Semaphorin-3C	0.350578
ATOH8	Protein atonal homolog 8	0.370948
SPTSSA	Serine palmitoyltransferase small subunit A	0.376402
SYNC	Syncoilin	0.379147
ALDH3A1	Aldehyde dehydrogenase, dimeric NADP-preferring	0.383731
COBLL1	Cordon-bleu protein-like 1	0.399284
DDIT4L	DNA damage-inducible transcript 4-like protein	0.40746
MPZL2	Myelin protein zero-like protein 2	0.408897
ADIRF	Adipogenesis regulatory factor	0.416268
CRIP2	Cysteine-rich protein 2	0.417072
ARL6IP5	PRA1 family protein 3	0.420077
ALPP	Alkaline phosphatase, placental type	0.423014
HEATR5A	HEAT repeat-containing protein 5A	0.427246
QPCT	Glutaminyl-peptide cyclotransferase	0.427804
CLIC3	Chloride intracellular channel protein 3	0.431867
GLS	Glutaminase kidney isoform, mitochondrial	0.445419
TNS3	Tensin-3	0.458781
RHOBTB3	Rho-related BTB domain-containing protein 3	0.463897
IDH1	Isocitrate dehydrogenase [NADP] cytoplasmic	0.469876
C14orf132	Uncharacterized protein C14orf132	0.476645
TIMP3	Metalloproteinase inhibitor 3	0.478399
CD14	Monocyte differentiation antigen CD14	0.488419
CXXC5	CXXC-type zinc finger protein 5	0.491275
SUMF1	Sulfatase-modifying factor 1	0.492325

## DISCUSSION

To our knowledge, this is the first study reporting KSHV infection and/or viral latent proteins can down-regulate HPV16 E6 and E7 expression from cervical cancer cells. We also have found the underlying mechanism is through the down-regulation of at least one cellular microRNA, miRNA129-5p, although some other mechanisms are possibly involved as well. Interestingly, the down-regulation of E6 and E7 expression usually causes the suppression of cervical cancer cell proliferation and the promotion of cancer cell apoptosis [19–22]. However, we do not observe that KSHV infection can affect SiHa cell proliferation or viability, implying that some KSHV-related factors (viral or host) may compensate the effects of down-regulation of HPV16 E6 and E7 on cancer cell growth. Instead, we have found that KSHV infection greatly reduced SiHa cell invasiveness when compared to the control mock cells (data not shown). It remains unclear whether this down-regulation of E6 and E7 by KSHV is cell-line or HPV subtype specific, although we have reported that KSHV can establish latent infection within HPV18-integrated HeLa cells as well [23]. So we will test the susceptibility of other HPV+ cervical cancer cell-lines to KSHV infection, replication and their impacts on HPV-encoded oncoproteins such as E6 and E7.

One remaining question is the mechanisms through which miRNA129-5p inhibiting E6 and E7 expression. Zhang *et al*. have reported that the transcription factor SP1 is a direct downstream target of miR-129-5p in Hela cells, and SP1 expression is down-regulated significantly by over-expressed miR-129-5p [19]. Interestingly, the upstream regulatory regions of HPV-18 genes contain the SP1 binding site, which have been shown to determine E6 and E7 expression [24, 25]. So future work will try to determine whether the similar mechanisms are present in SiHa cells, or whether E6 and E7 are direct targets by miR-129-5p. Another interesting question is how KSHV infection can manipulate miR-129-5p expression in SiHa cells. In fact, KSHV infection or viral proteins have been found to regulate a variety of cellular microRNAs expression [26]. For instance, Tsai and colleagues have reported that KSHV-encoded K15 protein, minor form named as K15M, can induce cell migration and invasion through the up-regulation of cellular miR-21 and miR-31 via its conserved Src-Homology 2 (SH2)-binding motif [27]. KHSV infection and ectopic expression of vFLIP can suppress the expression of one chemokine receptor, CXCR4, through the up-regulation of cellular miR-146a expression, a miRNA that is known to bind to the 3′UTR of CXCR4 mRNA [28].

In the present study, we have found that KSHV *de novo* infection can induce some inflammatory cytokines/chemokines production from SiHa cells. Interestingly, one of these factors, CXCL1 (also named as growth regulated oncogene 1, GRO-1), its serum levels were significantly higher in patients with cervical squamous cell carcinoma (CSCC) when compared with patients with cervical intraepithelial neoplasia (CIN) and the healthy controls [29]. However, these up-regulated inflammatory cytokines/chemokines may promote the recruitment of immune cells such as neutrophils and macrophages, enhance local inflammatory response, and finally facilitate attacking infected cells and/or the clearance of viruses. Another interesting finding in our study is that KSHV infection up-regulates a series of IFN-induced genes expression from SiHa cells. We actually have observed similar responses within KSHV-infected human primary oral fibroblasts [30]. As we know, high-risk HPV infection down-regulates IFNα-inducible gene expression, for example, the HPV16 E6 and E7 oncoproteins interact directly with the components of the IFN signaling pathways [31]. Since IFN has been widely used in the treatment of CIN and cervical cancer, the down-regulation of E6 and E7 expression may be responsible for the positive clinical outcomes observed with IFN treatment [19]. Therefore, our data suppose that KSHV co-infection may arouse IFN signaling pathway activities to accelerate virus clearance by host immune system. Taken together, our data have provided possible explanations for very low detection rate of KSHV shedding as well as of KSHV/HPV co-infection in cervical samples and/or cervical cancer cells.

## MATERIALS AND METHODS

### Cell culture and KSHV purification/infection

Body cavity-based lymphoma cells (BCBL-1, KSHV^+^/EBV^neg^) were kindly provided by Dr. Dean Kedes (University of Virginia) and maintained in RPMI 1640 medium (Gibco) with supplements as described previously [32]. SiHa cells were purchased from ATCC and maintained in Eagle's Minimum Essential Medium (ATCC) supplemented with 10% FBS. All cells were incubated at 37°C in 5% CO_2_. All experiments were carried out using cells harvested at low passages (< 20). To obtain KSHV for infection experiments, BCBL-1 cells were incubated with 0.6 mM valproic acid for 6 days, and purified virus was concentrated from culture supernatants and infectious titers were determined as described previously [33].

### Human cytokine/chemokine array

The human cytokine/chemokine array was performed by using Proteome Profiler^TM^ Array (R&D Systems) which contains 36 different human cytokines according to the manufacturers’ instructions. The density of dot-blot was scanned and quantified by using the ImageJ software.

### Microarray

Microarray analysis was performed and analyzed at the Stanley S. Scott Cancer Center's Translational Genomics Core at LSUHSC. Total RNA was isolated using Qiagen RNeasy kit (Qiagen), and 500 ng of total RNA was used to synthesize dscDNA. Biotin-labeled RNA was generated using the TargetAmp-Nano Labeling Kit for Illumina Expression BeadChip (Epicentre), and hybridized to the HumanHT-12 v4 Expression BeadChip (Illumina) at 58°C for 16 h. The chip was washed, stained with streptavadin-Cy3, and scanned with the Illumina BeadStation 500 and BeadScan. Using the Illumina's GenomeStudio software, we normalized the signals using the “cubic spline algorithm” that assumes that the distribution of transcript abundance is similar in all samples. The background signal was removed using the “detection *p*-value algorithm” to remove targets with signal intensities equal or lower than that of irrelevant probes (with no known targets in the human genome but thermodynamically similar to the relevant probes). The microarray experiments were performed twice for each group and the average values were used for analysis. The enrichment analysis were performed using the MetaCore Software (Thompson Reuters). The microarray original data have been submitted to Gene Expression Omnibus (GEO) database (Accession number: GSE90039).

### Cell proliferation and apoptosis assays

Cell proliferation was measured by using the WST-1 assays (Roche) according to the manufacturers’ instructions. Flow cytometry was used for quantitative assessment of apoptosis using the FITC-Annexin V/propidium iodide (PI) Apoptosis Detection Kit I (BD Pharmingen).

### Immunoblotting

Total cell lysates (20 μg) were resolved by 10% SDS–PAGE, transferred to nitrocellulose membranes, and immunoblotted with antibodies for K8.1 (ABI) and β-Actin (Sigma) for loading controls. Immunoreactive bands were identified using an enhanced chemiluminescence reaction (Perkin-Elmer), and visualized by autoradiography.

### Immunofluorescence

Cells were incubated in 1:1 methanol-acetone at −20°C for fixation and permeabilization, then with a blocking reagent (10% normal goat serum, 3% bovine serum albumin, and 1% glycine) for an additional 30 minutes. Cells were then incubated for 1 h at 25°C with 1:1000 dilution of a rat anti-LANA monoclonal antibody (ABI) followed by 1:200 dilution of a goat anti-rat secondary antibody conjugated to Texas Red (Invitrogen). For identification of nuclei, cells were subsequently counterstained with 0.5 mg/mL 4′,6-diamidino-2-phenylindole (DAPI; Sigma) in 180 mM Tris-HCl (pH 7.5). Slides were washed once in 180 mM Tris-HCl for 15 min and prepared for visualization using a Leica TCPS SP5 AOBS confocal microscope.

### Transfection assays

For plasmid transfection, SiHa were transfected with control vector pcDNA3.1, pcDNA3.1-LANA (pcLANA) or pcDNA3.1-vFLIP (pcvFLIP) [34, 35] in 12-well plates for 48 h using Lipofectamine 2000 (Invitrogen) according to the manufacturer's instruction. Transfection efficiency was determined through co-transfection of a lacZ reporter construct and quantified as described previously [36].

### qRT-PCR

Total RNA was isolated using the RNeasy Mini kit (QIAGEN), and cDNA was synthesized from equivalent total RNA using a SuperScript III First-Strand Synthesis SuperMix Kit (Invitrogen) according to the manufacturer's instructions. Primers used for amplification of target genes are listed in Supplementary Table 1. Amplification was carried out using an iCycler IQ Real-Time PCR Detection System, and cycle threshold (Ct) values were tabulated in duplicate for each gene of interest in each experiment. “No template” (water) controls were used to ensure minimal background contamination. Using mean Ct values tabulated for each gene, and paired Ct values for β-actin as a loading control, fold changes for experimental groups relative to assigned controls were calculated using automated iQ5 2.0 software (Bio-rad). For amplification of human miRNAs, cDNA was synthesized using the Taqman miRNA RT kit (Applied Biosystems), and qPCR was performed using the Taqman MicroRNA Assays kit (Applied Biosystems) and a 7500 Real Time PCR System. Fold changes for microRNA were calculated using paired Ct values for RNU6B as recommended by the manufacturer (Applied Biosystems). The mirVana™ miRNA inhibitors for blocking miR129-5p or miR331-3p activities and the control were purchased from Invitrogen, and used according to the manufacturer's instructions.

### Statistical analysis

Significance for differences between experimental and control groups was determined using the two-tailed Student's *t*-test (Excel 8.0), and *p* values < 0.05 or < 0.01 were considered significant or highly significant, respectively.

## SUPPLEMENTARY FIGURES AND TABLE


