# Supplementary material for: KSHV co-infection down-regulates HPV16 E6 and E7 from cervical cancer cells
Source: Oncotarget. 2017 Mar 15;8(22):35792–803. doi: 10.18632/oncotarget.16207 (PMC5482618; doi:10.18632/oncotarget.16207)
Supplement: Supplementary file 1 [file oncotarget-08-35792-s001.pdf]

KSHV co-infection down-regulates HPV16 E6 and E7 from cervical cancer cells

Supplementary Materials

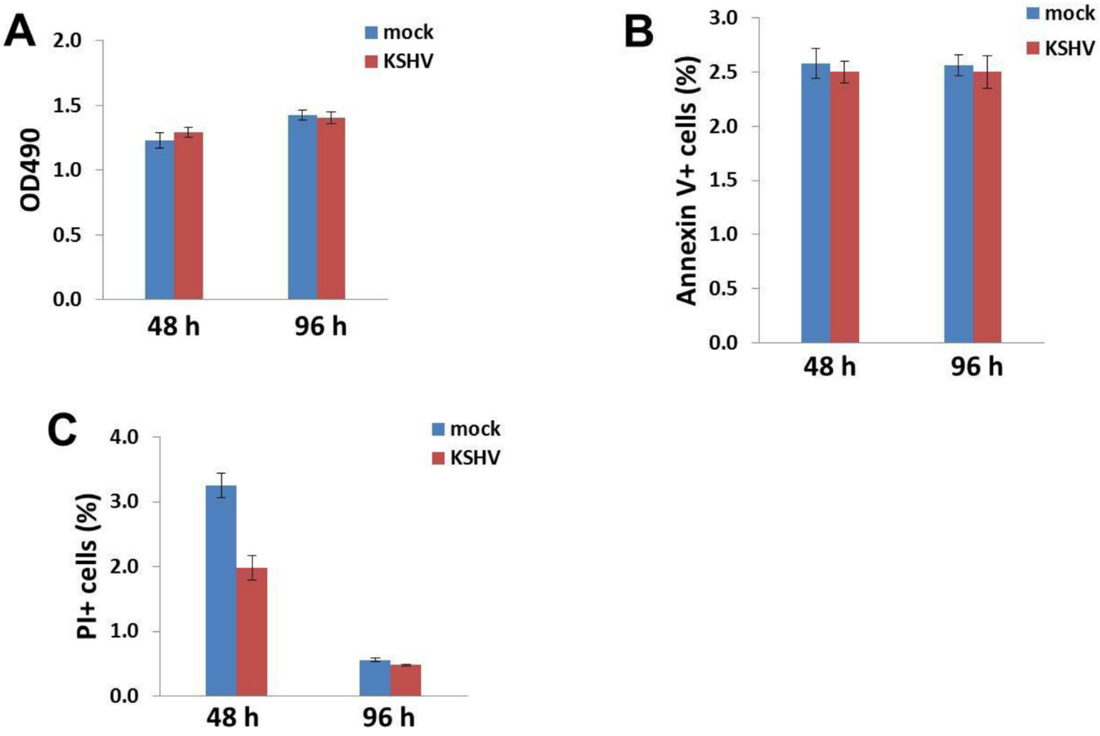

Supplementary Figure 1: KSHV *de novo* infection does not affect SiHa cell growth and viability. (**A**) SiHa were incubated with purified KSHV (MOI ~ 10), or medium control (mock) for 2 h. After cells were incubated for indicated additional time, cell proliferation was measured using the WST-1 assays. (**B–C**) Cell viability was measured by using flow cytometry as described in the Methods. Error bars represent the S.D. for 3 independent experiments.

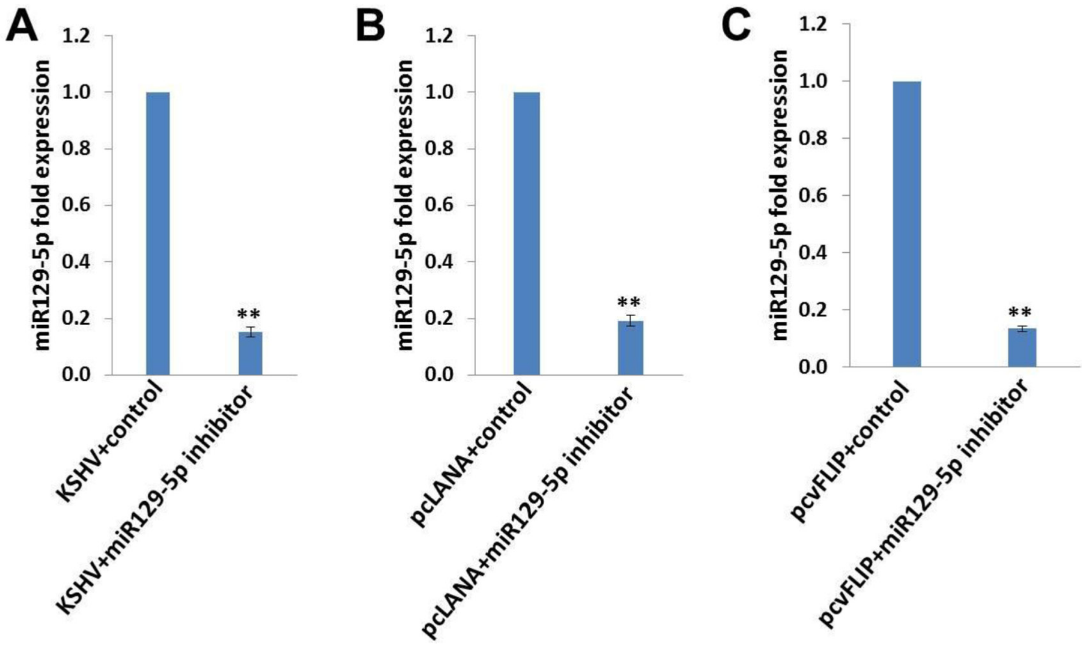

Supplementary Figure 2: Blocking miR129-5p activities by using specific miRNA inhibitor. (**A–C**) SiHa cells were transfected with control or miR129-5p inhibitor prior to KSHV infection or ectopic expression of LANA or vFLIP as described in the Methods, then miR129-5p transcripts were quantified by using qRT-PCR. The Error bars represent the S.D. for 3 independent experiments, **= $p < 0.01$.

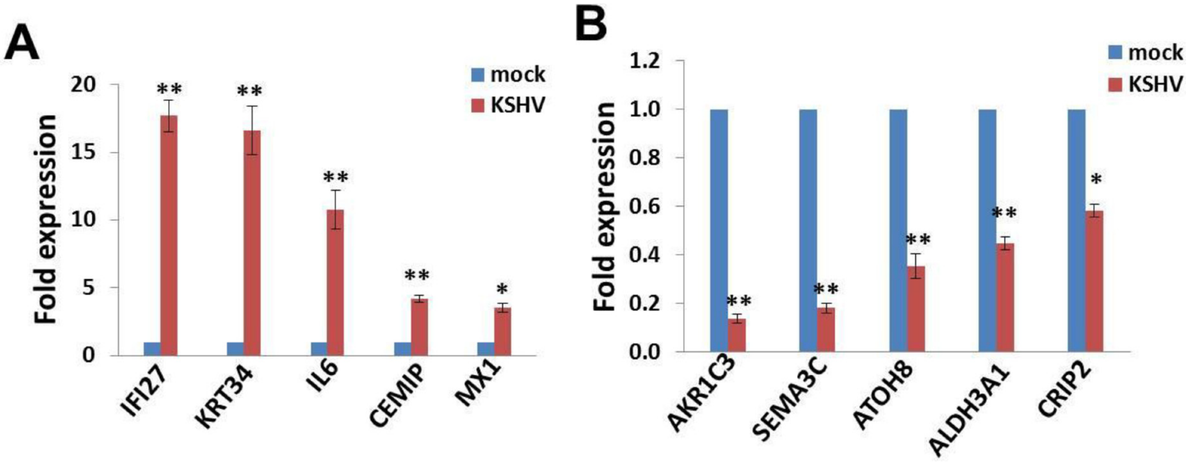

Supplementary Figure 3: Experimental validation of gene profile alterations in KSHV-infected SiHa cells. The transcriptional levels of 5 selected candidate genes that were up-regulated or down-regulated, respectively, in KSHV-infected SiHa cells from microarray data were validated by using qRT-PCR. The Error bars represent the S.D. for 3 independent experiments, *= $p < 0.05$, **= $p < 0.01$.

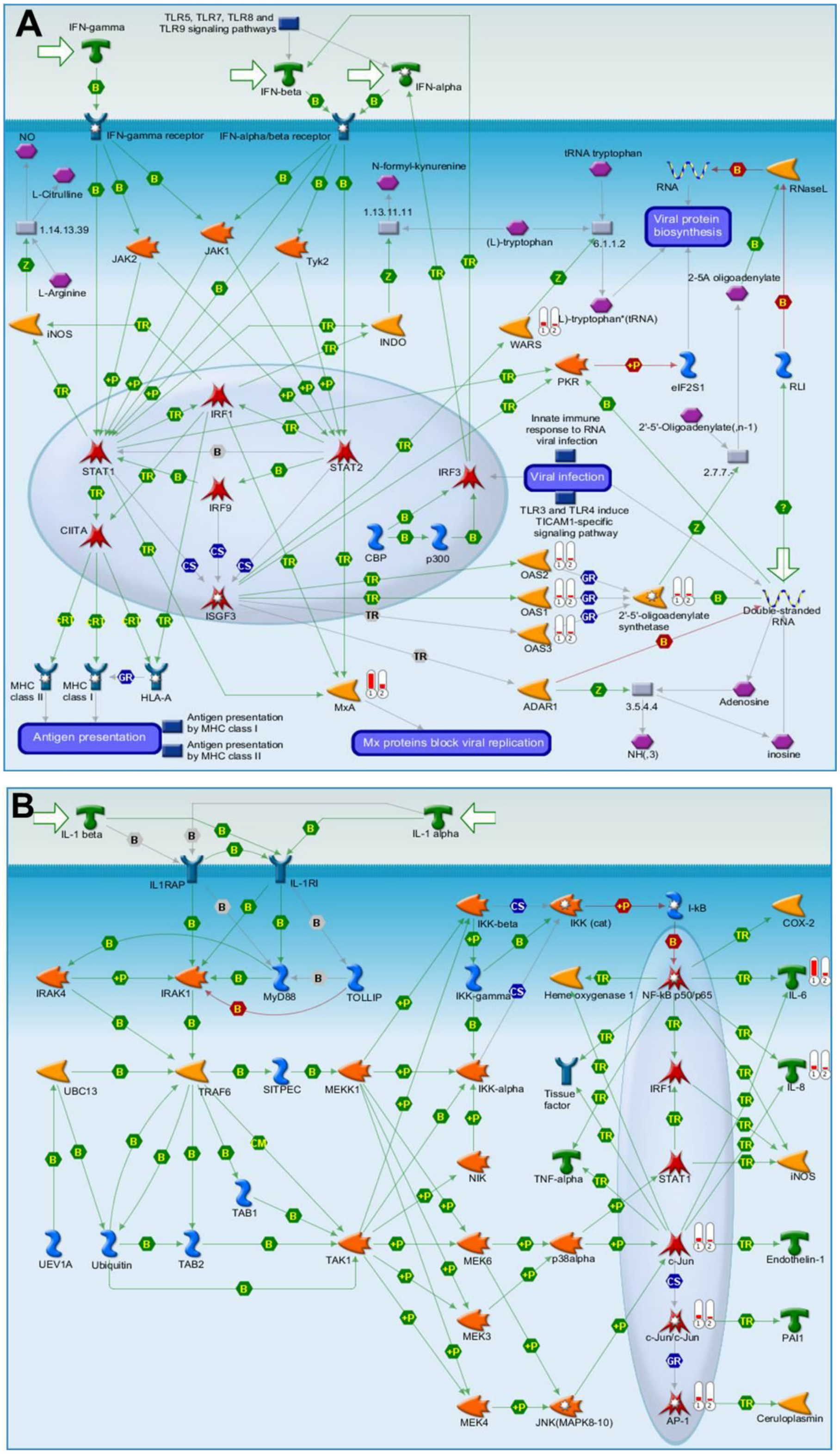

Supplementary Figure 4: The top 2 scored maps (maps with the lowest *p* value) based on the enrichment distribution sorted by gene set altered in KSHV-infected SiHa cells. (**A**) Immune response_Antiviral actions of Interferons. (**B**) Immune response_IL 1 signaling pathway. Experimental data from all files is linked to and visualized on the maps as thermometer like figures. Upward thermometers have red color and indicate upregulated signals and down ward (blue) ones indicate downregulated expression levels of the genes. Data was produced by the MetaCore Software (Thompson Reuters).

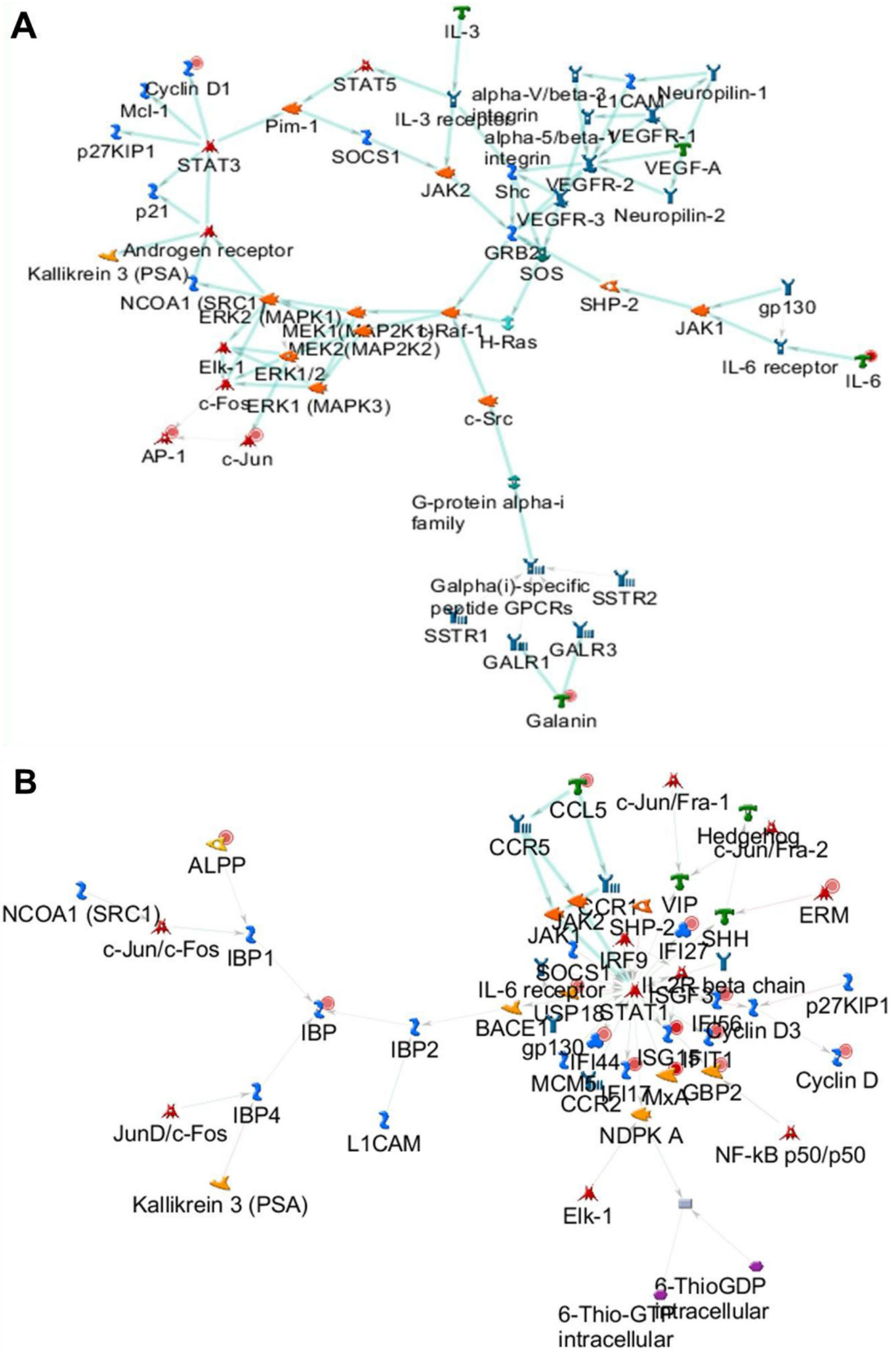

Supplementary Figure 5: The top 2 scored (by the number of pathways) AN networks from gene set altered in KSHV-infected SiHa cells. (**A**–**B**) Thick cyan lines indicate the fragments of canonical pathways. Upregulated genes are marked with red circles; downregulated with blue circles. The 'checkerboard' color indicates mixed expression for the gene between files or between multiple tags for the same gene. Data was produced by the MetaCore Software (Thompson Reuters).

Supplementary Table 1: Primer sequences for qRT-PCR

Gene	Sequences (5′→ 3′)
LANA	*sense* *TCCCTCTACACTAAACCCAATA*<br>*antisense* *TTGCTAATCTCGTTGTCCC*
RTA	*sense* *TAATGTCAGCGTCCACTCC*<br>*antisense* *TTCTGGCACGGTCAAAGC*
vGPCR	*sense* *CATCCGCTGCACTGTTAA*<br>*antisense* *GCTTTGTCCTCCTCACCA*
K8.1	*sense* *CACCACAGAACTGACCGATG*<br>*antisense* *TGGCACACGGTTACTAGCAC*
ORF57	*sense* *GGGTGGTTTGATGAGAAGGA*<br>*antisense* *CGCTACCAAATATGCCACCT*
IFI6	*sense* *TCGCTGATGAGCTGGTCTG*<br>*antisense* *GAGATACTTGTGGGTGGCGTAG*
IFI44	*sense* *CCTGCCGTTTATTCTGTG*<br>*antisense* *CGTTTACCAACTCCCTTC*
IFIT1	*sense* *CACCCACTTCTGTCTTACT*<br>*antisense* *ACATTCTTGCCAGGTCTA*
IFIT2	*sense* *ATACCAAACAATGCCTACC*<br>*antisense* *GAGCCACAGCGTGTCCTA*
IFIT3	*sense* *CAGATTGGGTGCTGCTAC*<br>*antisense* *GCTGATGGGATTGTTGCT*
IFITM1	*sense* *CAACATCCACAGCGAGAC*<br>*antisense* *TCACAGAGCCGAATACCA*
IFI27	*sense* *GCTACAGTTGTGATTGGAGGAG*<br>*antisense* *AATGGAGCCCAGGATGAA*
KRT34	*sense* *AGTCGGACATCAACAGCATA*<br>*antisense* *CGGTTAATTTCCACCAGAG*
IL-6	*sense* *GTCCAGTTGCCTTCTCCC*<br>*antisense* *GCCTCTTTGCTGCTTTCA*
CEMIP	*sense* *CCATGAAAGGCTGTGAGA*<br>*antisense* *AGAGGTGGAAGAAGTGCTG*
MX1	*sense* *GACATTCGGCTGTTTACC*<br>*antisense* *CTTCCAGTGCCTTGATTT*
MX2	*sense* *ACCGCCATTCGGCACAGT*<br>*antisense* *TGCCCTTGGTTGGCTCCT*
ISG15	*sense* *TGGACAAATGCGACGAACC*<br>*antisense* *CCCGCTCACTTGCTGCTT*
AKR1C3	*sense* *TGCCTGTATTGGGATTTG*<br>*antisense* *CCTGCTCCTCATTATTGTAT*
SEMA3C	*sense* *TGCTGGAAACTGATAACC*<br>*antisense* *TCTGCCCTGATAGGAAAT*
ATOH8	*sense* *CGGCTGGCTGACCTTGACT*<br>*antisense* *GCTTCTTGGCACGTCCCTC*
ALDH3A1	*sense* *ATGAATGGAACGCCTACTATGA*<br>*antisense* *CCAGGTGCCAATGACGAG*
CRIP2	*sense* *CCGTGCTACGCCACCCTGTT*<br>*antisense* *TCGCCTTCCGCTCCTCTGCT*
HPV16-E6	*sense* *AGGAGCGACCCAGAAAGT*<br>*antisense* *GCATAAATCCCGAAAAGC*
HPV16-E7	*sense* *GGAGGATGAAATAGATGG*<br>*antisense* *ACAACCGAAGCGTAGAGT*
β-actin	*sense* *GGAAATCGTGCGTGACATT*<br>*antisense* *GACTCGTCATACTCCTGCTTG*
